# Biomarker-based risk assessment of dietary intervention in patients with coronary artery disease during cardiac rehabilitation—a quasi-experimental study

**DOI:** 10.3389/fnut.2025.1669931

**Published:** 2025-10-23

**Authors:** Mona Kotewitsch, Hubert Scharnagl, Dennis Köstler, Marc Teschler, René Garbsch, Hendrik Schäfer, Melina Waranski, Katalin Vereckei, Gereon Böll, Marcus E. Kleber, Alexander Dressel, Winfried März, Boris Schmitz, Frank C. Mooren

**Affiliations:** ^1^Department of Rehabilitation Sciences, Faculty of Health, University of Witten/Herdecke, Witten, Germany; ^2^DRV Clinic Königsfeld, Center for Medical Rehabilitation, Ennepetal, Germany; ^3^Clinical Institute of Medical and Chemical Laboratory Diagnostics, Medical University of Graz, Graz, Austria; ^4^Department of Medicine I (Cardiology, Angiology, Hemostaseology, Intensive Care), Medical Faculty Mannheim, University of Heidelberg, Mannheim, Germany; ^5^Synlab Human Genetics Laboratory, SYNLAB Holding Germany GmbH, Mannheim, Germany; ^6^Society for the Prevention of Cardiovascular Disease e.V. (DACH), Hamburg, Germany; ^7^Synlab Academy, SYNLAB Holding Germany GmbH, Mannheim, Germany

**Keywords:** cardiac rehabilitation, coronary artery disease, low-carbohydrate diet, low-fat diet, cardiovascular risk, dietary adherence

## Abstract

**Background and aims:**

Cardiac rehabilitation (CR) is integral to secondary prevention in coronary artery disease (CAD), incorporating exercise, medical optimization, and dietary interventions. While low-carbohydrate (low-carb) and low-fat diets may improve metabolic health, their comparative impact on cardiovascular risk in CR remains unclear. This study assessed the effects of low-carb and low-fat diets on cardiovascular risk, body composition, and major adverse cardiovascular and cerebrovascular events (MACCE) in CAD patients undergoing inpatient CR.

**Methods:**

In this quasi-experimental study, 313 CAD patients (56 ± 7 years, 20% women) participated in CR, adopting a low-carb (*n* = 58), low-fat (*n* = 136), or regular diet (*n* = 119, control). Dietary assignment was non-randomized and based on assisted patient self-selection. A biomarker-based score to estimate the 10-year cardiovascular mortality risk, bioelectrical impedance analysis, and laboratory parameters (HbA1c, lipids, inflammation markers) were assessed at baseline, discharge, and 6-month follow-up. Kaplan–Meier analysis was used to compare MACCE recorded for a mean of 470 ± 293 days.

**Results:**

During 3–4 weeks of CR, the 10-year cardiovascular mortality risk decreased by a mean of 3.7 ± 9.6%, with no difference between dietary groups (*p* = 0.8651). HbA1c improved in the low-carb group during CR compared to the low-fat and regular diet (−4.0 ± 6.6%), but the effect was not significant after adjustments for baseline HbA1c, diabetes prevalence, and medication (*p* = 0.168). Reductions in BMI, body fat, and visceral fat were recorded in the low-carb and low-fat group, compared to the control group (*p* ≤ 0.0001). Total cholesterol, LDL, and triglyceride levels also decreased in all groups during CR without significant differences (*p* ≥ 0.3957). MACCE incidence did not differ between the groups (*p* = 0.2).

**Conclusion:**

No additional immediate benefit in risk reduction during CR for low-carb or low-fat dietary interventions was detected. However, the low-fat and low-carb diet resulted in significantly greater reductions in BMI, body fat and visceral fat, with a tendency towards more stable effects over 6 months in the low-fat group. While glycemic control was improved in the low-carb group during inpatient CR, long-term adherence appeared challenging, particularly for diabetic patients as HbA1c levels re-increased during 6 months follow-up. Since no difference in MACCE was seen, the dietary interventions may be considered equally safe for CAD patients.

## Introduction

Cardiovascular disease (CVD) remains a leading cause of morbidity and mortality, with coronary artery disease (CAD) representing a substantial portion of this burden (1). The pathogenesis of CAD is multifactorial, involving a complex interplay of modifiable risk factors such as obesity, type 2 diabetes, dyslipidemia, sedentary behavior, and hypertension, as well as non-modifiable factors, including genetic predisposition and age (2). While advances in pharmacological treatments and interventional procedure have substantially improved outcomes, the persistent prevalence and recurrence rates of CAD underscore the critical need for effective secondary prevention strategies to attenuate disease progression and improve long-term prognosis (3, 4). Cardiac rehabilitation (CR) is a cornerstone of secondary prevention, offering a structured and multidisciplinary approach that integrates supervised exercise training, psychosocial support, and optimization of medical therapy, as well as comprehensive dietary intervention and guidance (5, 6). The primary goal of CR is to mitigate cardiovascular risk, enhance functional capacity, and improve quality of life (7). However, the response to CR interventions can vary significantly among patients, necessitating objective and sensitive methods to assess and stratify risk during and after rehabilitation (8–10). Biomarker-based risk assessment represents a promising advancement in the field, allowing for dynamic monitoring of physiological and metabolic responses to CR (11). These markers may provide insights into systemic inflammation and glycemic control for example, both relevant in the context of CAD.

Among the different components of CR, dietary interventions play a crucial role in addressing traditional cardiovascular risk factors such as obesity, dyslipidemia, hypertension, and insulin resistance, while also potentially affecting systemic inflammation and endothelial dysfunction (12, 13). Current dietary guidelines, such as the European Society of Cardiology (ESC) guidelines, emphasize adopting balanced, nutrient-dense diets, such as the Mediterranean diet and a general reduction in fat, to lower cardiovascular risk through improvements in lipids, blood-pressure, body weight, and glycemic control (14). However, emerging evidence highlights the benefits and trade-offs also of more specific dietary strategies, including low-carbohydrate (low-carb) diets, within the context of CR. Low-carb diets, characterized by reduced carbohydrate intake, have shown favorable effects on weight management, glycemic control, and lipid parameters such as high-density lipoprotein cholesterol (HDL-C) and triglycerides (13, 15, 16). However, their long-term effect and cardiovascular safety in high-risk populations remain inconclusive and limited by small sample sizes and short-duration follow-up (15, 17). Conversely, low-fat diets emphasize reductions in fat intake, particularly saturated fat, combined with increased consumption of complex carbohydrates and lean protein. These diets have been associated with improvements in lipid profiles and systemic inflammation (16, 18). Despite their potential, adherence to low-fat diets poses challenges, particularly among individuals accustomed to higher-fat eating patterns. Despite the growing interest in these dietary paradigms in the field of CR in particular and in secondary prevention in general, the comparative impact of low-carb and low-fat diets on cardiovascular risk reduction remain poorly understood. Furthermore, prospective studies with adequate long-term follow-up periods to investigate the adherence to dietary patterns are missing from the field. This knowledge gap limits the ability to provide evidence-based dietary recommendations tailored to individual risk profiles and therapeutic needs specifically during CR.

### Objective

This quasi-experimental study aimed to evaluate the effects of two controlled dietary strategies, a “low-carb” diet and a “low-fat” diet, on the cardiovascular risk profile of CAD patients during inpatient CR. During a six-months follow-up period, the long-term effects of the dietary intervention were investigated. A biomarker-based risk assessments was used to compare the efficacy of these dietary interventions in reducing the 10-year CVD mortality risk.

## Materials and methods

### Study design

An interventional study was conducted between 2021 and 2024 at medical rehabilitation center Clinic Königsfeld Germany, to investigate the effect of a low-carb diet and a low-fat diet on the cardiovascular risk profile in patients with CAD undergoing guideline-based inpatient phase II CR (Clinical Trials: NCT05461729) (2, 7, 14). The study received ethical approval from the ethics committee of the University of Witten/Herdecke (approval number: 115/2020) and adhered to the principles of the Declaration of Helsinki for research involving human participants. All participants provided written informed consent prior to inclusion. Comprehensive clinical assessments were conducted at predefined time points and included blood sampling, anthropometric measurements, body composition analysis using bioelectrical impedance analysis (BIA), and blood pressure measurements. Patient medical histories, covering disease severity, comorbidities, prior interventions, and medications, were retrieved from electronic health records by clinical staff at the patients’ request and were provided by the patient after CR. Data collection occurred at three distinct time points: at admission to inpatient CR (T0), prior to discharge (3–4 weeks post-admission, T1), and 6 months (T2) after discharge. Additional data on mortality, rehospitalization, and subsequent medical interventions were collected through telephone interviews with patients and their treating physicians.

### Inclusion criteria

Patients with CAD, including those who had experienced a myocardial infarction (MI; STEMI/NSTEMI) and/or undergone angioplasty, percutaneous coronary intervention (PCI), or coronary artery bypass graft (CABG) surgery, who were participating in inpatient phase II CR, and who demonstrated a readiness to adopt dietary modifications were eligible to participate.

Patients who were unable/ unwilling to provide informed consent or had medical conditions that precluded participation in CR, such as unstable coronary or cerebrovascular conditions or acute infections were excluded. Patients who underwent ambulant CR were also not eligible. Only data of patients with complete biomarker assessment at both time points, T0 and T1, was analyzed.

### Laboratory parameters

This study used the cardiac rehabilitation biomarker score (CRBS), developed based on extensive empirical research spanning decades on biomarkers related to CAD, as detailed in previous studies (11, 19, 20). The CRBS was designed through a comprehensive analysis of over 200 biomarkers to identify those most predictive of vascular risk. By incorporating age, sex, smoking status, and specific laboratory parameters related to different physiological domains, the CRBS estimates a 10-year mortality risk. The selected biomarkers include hemoglobin A1c (HbA1c), N-terminal pro-brain natriuretic peptide (NT-proBNP), high-sensitivity troponin I (hsTnI), cystatin C, and high-sensitivity C-reactive protein (hsCRP). Venous blood samples were collected in the morning under standardized, non-fasting conditions. Blood was centrifuged at 3000 g for 10 min within 30 min of venipuncture, and serum was aliquoted into 1 ml portions before being stored at −80 °C for subsequent analysis. HbA1c was measured from EDTA whole blood at an accredited laboratory (SYNLAB MVZ Leverkusen, Germany) using standard protocols. Serum samples were analyzed anonymously at the Clinical Institute of Medical and Chemical Laboratory Diagnostics, Medical University of Graz, using validated assays and equipment. Lipid profiles, including total cholesterol, HDL cholesterol, LDL cholesterol, and triglycerides, were quantified enzymatically with reagents from DiaSys (Holzheim, Germany). NT-proBNP and hsTnI were measured via immunochemiluminescence assays supplied by Abbott Diagnostics (Abbott Park, IL, USA). Interassay coefficients of variation (CVs) for NT-proBNP were 2.3, 3.1, and 2.8% at mean concentrations of 153, 525, and 4,850 pg/ml, respectively, while those for hsTnI were 5.4, 5.8, and 6.6% at mean concentrations of 21, 509, and 17,440 pg/ml. Cystatin C was assessed using immunoturbidimetry (DiaSys, Holzheim, Germany), with interassay CVs of 1.7 and 1.8% at concentrations of 1.7 and 1.8 mg/L, respectively. CRP levels were measured using a particle-enhanced immunoturbidimetric assay from DiaSys, with interassay CVs of 3.5 and 2.5% at concentrations of 15.4 and 73.8 mg/L. Lipoprotein(a) [Lp(a)] was determined using immunoturbidimetry, also with DiaSys reagents and standards. Analyses were performed using a Beckman Coulter AU680 analyzer (Beckman Coulter, Brea, CA, USA) for lipids, cystatin C, and CRP, while an Abbott Architect i2000SR analyzer was employed for NT-proBNP and hsTnI measurements.

### Major adverse cardiac and cerebrovascular events (MACCE)

MACCEs were defined as all-cause death, the incidence of cardiovascular or cerebrovascular events or unplanned medical interventions due to cardiovascular complications during the follow-up period of up to 3 years post-inpatient rehabilitation. These events included stent placement, in-stent restenosis, bypass surgery, defibrillator or pacemaker implantation, MI, and stroke.

### Rehabilitation process

During the inpatient rehabilitation program, patients participated in various physical therapies tailored to their needs based on initial stress test results, following current guideline recommendations as described (14). The interventions included aerobic group exercises such as ergometer training, medical training therapy, aqua fitness, terrain walking, circuit training, and resistance training. Prescription of physical exercise training was equal for all patients and did not differ by dietary intervention (Supplementary Table 1).

### Bioelectrical impedance analysis (BIA)

Body composition, including body mass index (BMI), body fat mass, skeletal muscle mass, visceral fat, and waist-to-hip ratio, was assessed at each time point using direct-segmental multifrequency bioelectrical impedance analysis (DSM-BIA, Inbody720, Biospace, Seoul, Korea). Patients with pacemakers, life vests, or similar devices were excluded from BIA measurements.

### Dietary intervention

During inpatient CR, all patients were provided with full board meals (breakfast, lunch and dinner) prepared by the rehabilitation center, based on the recommendations of the German Nutrition Society (Deutsche Gesellschaft für Ernährung, DGE) (21–23). The dietary regimens were planned isocaloric, providing an energy intake of approximately 1,800 kcal per day. For patients following a diet reduced in carbohydrates (“low-carb” diet), the macronutrient distribution was planned to consist of ≤25% carbohydrates, 45% fats, and 30% proteins, whereas the diet reduced in fats (“low-fat” diet) was composed of 55% carbohydrates, ≤30% fats, and 15% proteins. For patients who did not opt for one of the two intervention diets, a recommended standard diet was provided with a macronutrient composition of approximately 40% of total energy from carbohydrates, 40% from fat, and about 20% from protein. As part of the general CR program, each patient attended a standardized nutrition lecture aimed at enhancing awareness of healthy dietary practices for cardiovascular health. The lectures were provided by certified nutritional experts of the CR center. Patients who expressed a readiness to adopt dietary modifications were offered a choice between the low-carb diet and low-fat diet. Patients then participated in supplementary seminars and received personalized consultations tailored to their chosen dietary regimen. These sessions included customized educational materials to reinforce dietary recommendations. The type and quantity of meals were standardized to ensure consistency. Lunch was plated and served directly by the kitchen staff, while breakfast and dinner were self-served. To facilitate appropriate food choices during self-service meals, patients were trained using model plates illustrating portion sizes and nutrient compositions specific to their assigned dietary plan.

### Dietary adherence control

To assess dietary adherence, patients completed a daily dietary questionnaire documenting their food choice (e.g., standard, low-carb, or low-fat) for each meal (breakfast, lunch, dinner) over the 3 to 4 weeks of CR. Based on the cumulative questionnaire data, patients` data was analyzed as treated if more than 60% of their reported intake aligned with the specific dietary pattern over the course of their rehabilitation stay. This threshold was chosen in accordance with prior work in dietary trials, reflecting a meaningful adherence while preserving sufficient sample size (24, 25). The exact corresponding macronutrient intake was calculated based on detailed menu plans provided by the kitchen staff.

### Comparator score calculation

The Framingham score was calculated based on a multivariable model incorporating sex, age, total and HDL cholesterol, systolic blood pressure, use of antihypertensive medication, smoking status, and the presence of diabetes, as previously described (26, 27).

### Statistical analysis

Statistical analyses were conducted using SPSS (version 28, IBM, Armonk, USA), R (version 4.3.0, https://www.r-project.org) (28), and GraphPad Prism (version 10, GraphPad Software, Boston, USA). Continuous variables were reported as means ± standard deviation (SD) or medians with 95% confidence intervals (CI), while categorical variables were presented as absolute numbers (n) and percentages (%). Subgroup analyses were performed based on sex (male, female), age groups (<50, 50–60, >60), presence of diabetes mellitus, disease severity (number of affected coronary vessels), and left ventricular function (LVEF reduction, no LVEF reduction). To account for multiple testing in the subgroup analyses, *p*-values were adjusted using the Benjamini-Hochberg false discovery rate (FDR; q < 0.05). Responder analysis was conducted to identify individuals who demonstrated a clinically relevant change in BMI during inpatient rehabilitation. Patients were categorized as responders or non-responders based on a typical error (TE) method, analogous to previous analyses (29). The TE was calculated using the formula: $\mathrm{TE}=\text{SDdiff}/\sqrt{2}$ , where SD_diff_ represents the standard deviation of the difference in BMI between T0 and T1. Participants were classified as responders if their relative BMI reduction exceeded the TE, indicating a true change beyond expected measurement variability. The proportion of responders was calculated for each dietary group and differences between groups were analyzed using Chi-square tests. Group differences over time were analyzed using two-way repeated measures ANOVA, or mixed-effects models in case of missing data. ANCOVA was employed to examine biomarker score differences across groups while controlling for age, BMI, and HbA1c. Kaplan–Meier survival curves were generated using the R package “survminer” (version 0.4.9.999) (30) to compare MACCE rates among patients grouped by dietary patterns (low-carb/low-fat vs. standard diet). A Cox proportional hazards regression model was generated using “survival” (version 3.2–13) with cardiovascular risk as estimated by the CRBS as a covariate to adjust for group differences at T1. The proportional hazards assumption was evaluated using Schoenfeld residuals and a post-hoc power calculation was performed using a two-sided log-rank test (α = 0.05). In addition, a fully adjusted sensitivity model was calculated including age, sex, diabetes mellitus, CAD extent, history of MI or CABG, HbA1c, LDL-C, and BMI (at T1) as covariates. All tests were two-sided, and a *p*-value less than 0.05 was considered statistically significant.

## Results

### Baseline characteristics

**Figure 1:**
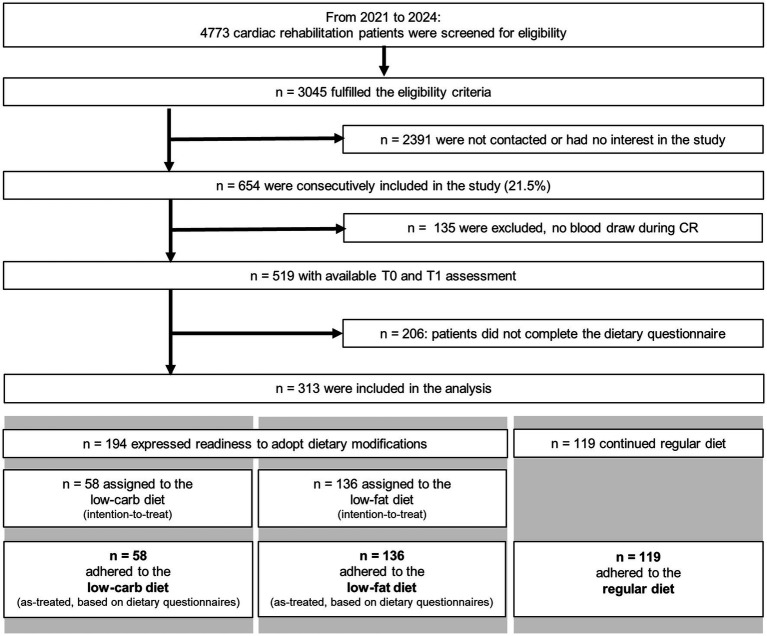
Study flow-chart.

A total of 313 CAD patients (20% women) were included and provided a full dietary questionnaire allowing the allocation into one of the three dietary interventions: low-fat diet (*n* = 136), low-carb diet (*n* = 58), and regular diet (*n* = 119) (Figure 1). The mean age of the study population was 56 ± 7 years, with no significant difference between the groups (*p* = 0.068; Table 1). Patients in both the low-fat and low-carb groups exhibited significantly higher weight (low-fat, 97 ± 21 kg; low-carb, 100 ± 18 kg), BMI (low-fat, 30.9 ± 5.3 kg/m^2^; low-carb, 32.6 ± 5.1 kg/m^2^), body fat mass (low-fat, 32.7 ± 13.3 kg; low-carb, 36.8 ± 10.9 kg), and visceral fat (low-fat, 156.6 ± 62.4 cm^2^; low-carb, 178.4 ± 51.0 cm^2^) compared to the regular diet group (weight, 88 ± 16 kg; BMI, 28.4 ± 4.2 kg/m^2^; body fat mass, 25.9 ± 9.9 kg; visceral fat, 125.9 ± 51.1 cm^2^; all *p* ≤ 0.001). Additionally, the prevalence of obesity (*p* = 0.005) and diabetes mellitus (*p* < 0.001) was significantly higher in the low-carb group (45% and 41%, respectively). However, there were no significant differences between the groups concerning CAD severity, history of MI (within the last 12 months), or previous cardiac interventions. In terms of laboratory parameters, HbA1c levels were significantly elevated in the low-carb group (6.2 ± 1.0%) compared to the low-fat (5.8 ± 0.6%) and regular diet groups (5.8 ± 0.8%; *p* = 0.002), while results of other laboratory values were comparable between groups and within the reference range. Cardiopulmonary exercise capacity was reduced to ~75% of reference and comparable between groups (Table 1). Of note, comparative analysis of baseline characteristics and medication profiles between patients with and without 6-months follow-up data did not reveal significant differences across dietary groups (all *p* ≥ 0.3).

**Table 1:** Patients’ baseline characteristics by dietary group.

Group	Overall (*n* = 313)	Low-carb (*n* = 58)	Low-fat (*n* = 136)	Regular diet (*n* = 119)	*p*-value
Anthropometric data					
Age (years)	56 ± 7	54 ± 6	56 ± 7	56 ± 8	0.068
Sex, *n* (%)					0.053
Female	62 (20)	18 (31)	25 (18)	19 (16)	
Male	251 (80)	40 (69)	111 (82)	100 (84)	
Height (cm)	176 ± 9	175 ± 10	177 ± 9	176 ± 8	0.574
Weight (kg)	94 ± 19	100 ± 18^#^	97 ± 21^§^	88 ± 16	<0.001
BMI (kg/m^2^)	30.3 ± 5.1	32.6 ± 5.1^#^	30.9 ± 5.3^§^	28.4 ± 4.2	<0.001
Body Fat Mass (kg)	30.9 ± 12.4	36.8 ± 10.9^#^	32.7 ± 13.3^§^	25.9 ± 9.9	<0.001
Skeletal Muscle Mass (kg)	35.5 ± 6.7	35.7 ± 7.1	35.8 ± 6.8	34.9 ± 6.4	0.490
Visceral Fat (cm^2^)	148.9 ± 59.7	178.4 ± 51.0^#^	156.6 ± 62.4^§^	125.9 ± 51.1	<0.001
Waist-to-Hip Ratio	1.0 ± 0.1	1.0 ± 0.1^#^	1.0 ± 0.1	1.0 ± 0.1	0.002
Clinical data					
Coronary artery disease, *n* (%)					0.371
One vessel disease	106 (34)	25 (43)	44 (32)	37 (31)	
Two vessel disease	103 (33)	13 (22)	48 (36)	42 (35)	
Three vessel disease	104 (33)	20 (35)	44 (32)	40 (34)	
Myocardial infarction, *n* (%)	200 (64)	39 (57)	92 (68)	70 (59)	0.296
STEMI	102 (33)	16 (28)	47 (35)	39 (33)	
NSTEMI	77 (25)	21 (36)	34 (25)	23 (19)	
Intervention, *n* (%)					0.479
PCI	283 (90)	52 (90)	126 (93)	105 (88)	
CABG	30 (10)	6 (10)	10 (7)	14 (12)	
Cardiac arrhythmia, *n* (%)					0.961
Acute	37 (12)	6 (10)	16 (12)	15 (13)	
Permanent	3 (1)	1 (2)	1 (1)	1 (1)	
Arterial hypertension, *n* (%)	216 (69)	47 (81)	92 (68)	77 (65)	0.079
LVEF, *n* (%)					0.274
Normal (>50%)	252 (80)	46 (79)	105 (77)	101 (85)	
Slightly reduced (41–50%)	33 (11)	5 (9)	15 (11)	13 (11)	
Moderately reduced (31–40%)	24 (8)	7 (12)	13 (10)	4 (3)	
Severely reduced (≤30%)	4 (1)	0	3 (2)	1 (1)	
Endocrine, nutritional or metabolic diseases, *n* (%)					
Obesity	92 (29)	26 (45) ^#^	41 (30)	25 (21)	0.005
Diabetes mellitus	63 (20)	24 (41) ^#^	26 (19)	13 (11)	<0.001
Smoking status, *n* (%)					0.084
Active	53 (17)	5 (9)	22 (16)	26 (22)	
Laboratory parameter					
Total cholesterol (mg/dl)	144 ± 34	137 ± 29	144 ± 34	147 ± 35	0.138
LDL cholesterol (mg/dl)	71 ± 26	65 ± 22	71 ± 26	74 ± 27	0.110
Triglycerides (mg/dl)	122 ± 67	137 ± 75	115 ± 50	123 ± 77	0.107
HDL cholesterol (mg/dl)	44 ± 12	43 ± 13	44 ± 11	45 ± 11	0.667
c-reactive protein (mg/l)	4.4 ± 10.3	6.2 ± 17.7	3.1 ± 4.5	4.9 ± 9.9	0.127
HbA1c (%)	5.9 ± 0.8	6.2 ± 1.0*^,#^	5.8 ± 0.6	5.8 ± 0.8	0.002
hs-Troponin (pg/ml)	23 [95%-CI: 16–31]	20 [95%-CI: 8–32]	26 [95%-CI: 13–39]	22 [95%-CI: 10–34]	0.820
NT-proBNP (pg/ml)	606 [95%-CI: 504–709]	438 [95%-CI: 276–599]	688 [95%-CI: 493–883]	596 [95%-CI: 471–721]	0.222
Cystatin C (mg/l)	1.0 ± 0.2	1.0 ± 0.2	1.0 ± 0.2	1.0 ± 0.2	0.855
Vitamin D (ng/ml)	19.4 ± 13.3	20.3 ± 10.8	19.1 ± 10.2	19.3 ± 17.1	0.849
Physical performance					
Maximum workload (W)	135.1 ± 41.8	129.9 ± 40.4	141.7 ± 42.5	131.1 ± 41.0	0.161
VO_2_ at the anaerobic threshold (VT1)					
(ml/min/kg)	12.4 ± 2.8	11.9 ± 2.5	12.8 ± 3.1	12.1 ± 2.6	0.113
(% predicted)	50.0 ± 11.3	49.4 ± 11.0	50.7 ± 11.6	49.7 ± 11.2	
VO_2max_					
(ml/min/kg)	18.6 ± 4.4	18.0 ± 4.1	19.4 ± 4.8	18.0 ± 4.1	0.066
(% predicted)	74.2 ± 15.8	72.5 ± 14.2	76.3 ± 16.6	73.0 ± 15.4	
Medication					
ACE-inhibitor, *n* (%)	185 (59)	33 (57)	78 (57)	74 (62)	0.685
Anticoagulant, *n* (%)	262 (84)	50 (86)	115 (85)	97 (82)	0.684
Beta blocker, *n* (%)	263 (84)	46 (79)	115 (85)	102 (86)	0.537
Angiotensin II receptor blocker, *n* (%)	93 (30)	22 (38)	44 (32)	27 (23)	0.077
Calcium channel blocker, *n* (%)	64 (20)	12 (21)	27 (20)	25 (21)	0.973
Diuretic, *n* (%)	77 (25)	17 (29)	37 (27)	23 (19)	0.226
Diabetic medication, *n* (%)	42 (13)	21 (36)*	10 (7)	11 (9)	<0.001

Data is presented as mean ± SD, median with 95% CI, or *n* (%). BMI, Body Fat Mass, Skeletal Muscle Mass, Visceral Fat, and Waist-to-Hip Ratio were obtained through bioelectrical impedance analysis (BIA) and were available for 301 patients (patients with pacemakers, life-vests, etc. are not eligible for BIA). Myocardial Infarction is only listed if it had occurred within the previous 12 months before the start of CR. Diabetic medication includes metformin and insulin. ACE, angiotensin-converting enzyme; STEMI, ST-elevation myocardial infarction; NSTEMI, non-ST-elevation myocardial infarction; PCI, percutaneous coronary intervention; CABG, coronary artery bypass graft; LVEF, left ventricular ejection fraction; VO_2max_, maximal oxygen uptake during symptom-limited cardiopulmonary exercise testing. % Predicted values are corrected for sex, age, and body surface are. *p* values indicate between-group comparison by ANOVA or Chi-squared test. *Significant between low-carb and low-fat, ^#^significant between low-carb and regular diet, ^§^significant between low-fat and regular diet with *p* < 0.05.

### Implementation of dietary interventions

The dietary interventions were implemented in a controlled environment within the CR center to ensure adherence to predefined macronutrient uptake. Based on the weekly menu plans and provided recipes, the mean energy intake from lunch per week was calculated with 3,396 ± 35 kcal for the low-fat diet, 3,706 ± 162 kcal for the low-carb diet, and 4,441 ± 268 kcal for the regular diet, with the regular diet being significantly higher than both the low-fat and low-carb diet (both *p* ≤ 0.002). Based on the assumption that lunch accounted for one-third of total daily energy intake (≈600 kcal/day, 4,200 kcal/week), this equated to deviations from the planned meals by −19.1% for the low-fat diet, −11.8% for the low-carb diet, and +5.7% for the regular diet. Macronutrient distribution analysis confirmed that the low-fat diet was implemented as planned with a significantly lower fat (25.4%; 95.8 ± 6.1 g/week) and higher carbohydrate (44.8%; 380.7 ± 37.4 g/week) intake, while the low-carb diet exhibited the opposite pattern (fat, 47.1%, 194.1 ± 21.9 g/week; carbohydrate, 20.7%, 191.9 ± 22.1 g/week). Protein intake was consistent across all diets, with the highest values observed in the low-carb group (low-carb, 44.5%, 277.1 ± 21.2 g/week; low-fat, 30.2%, 226.9 ± 37 g/week; regular diet, 26.4%, 231.7 ± 24.6 g/week.

### Changes in body composition by dietary intervention during CR

**Figure 2:**
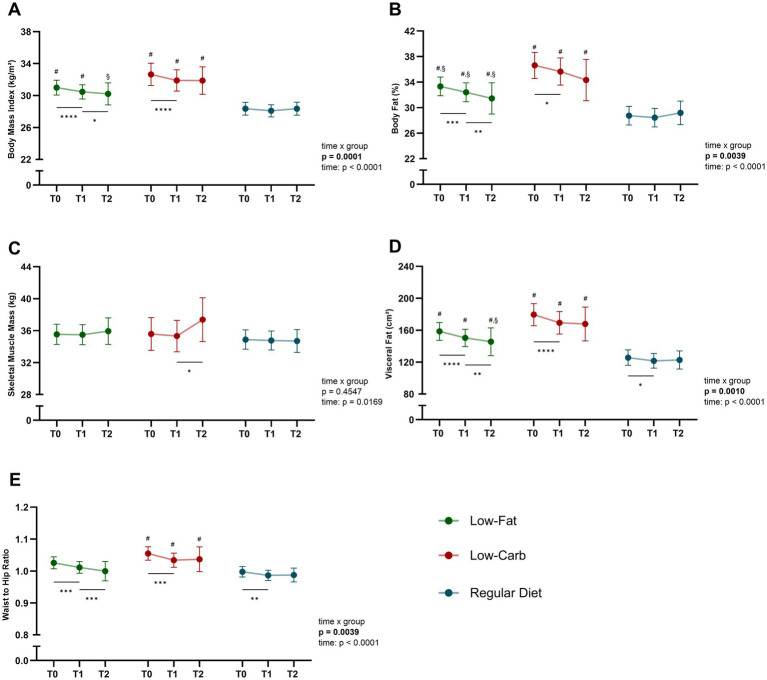
Changes in body composition across dietary interventions. Significant improvements in body composition during inpatient CR across all dietary groups (low-fat, low-carb, and regular diet) were seen. Data is presented for CAD patients grouped by dietary intervention in terms of: **(A)** body mass index (BMI), **(B)** body fat percentage, **(C)** skeletal muscle mass, **(D)** visceral fat, and **(E)** waist-to-hip ratio. Data is presented as mean with 95% confidence interval (CI). *Significant within-group effects, ^#^significant between low-fat/ low-carb and regular diet, §significant between low-fat and low-carb with *p* < 0.05. ****p < 0.0001, ***p < 0.001, **p < 0.01, *p < 0.05.

Reductions in BMI, body fat percentage, and visceral fat were observed between T0 and T1 across dietary groups, with significant differences in the magnitude of change between groups (time × group, all *p* ≤ 0.0050; Figure 2). BMI decreased significantly in the low-fat (−1.6 ± 2.1%, *p* < 0.0001) and low-carb group (−2.3 ± 1.6%, p < 0.0001), while no significant reduction in the regular diet group was detected (−0.8 ± 1.8%, *p* = 0.0531; Figure 2A). Responder analysis revealed a significant difference for change in BMI between dietary groups (responder, low-carb, 67%; low-fat, 53%; regular diet, 35%; *p* < 0.0001), with a significant difference observed between the low-carb and regular diet groups. Consistently, body fat percentage was significantly reduced in the low-carb (−2.8 ± 4.9%, *p* = 0.0174) and low-fat (−2.6 ± 5.4%, *p* = 0.0006) group, while no significant change in the regular diet group was observed (−1.0 ± 4.9%, *p* = 0.4122; time × group, *p* = 0.005; Figure 2B). Visceral fat decreased significantly in all groups, with the greatest reduction in the low-carb group (−6.1 ± 5.7%, *p* < 0.0001) and low-fat group (−4.8 ± 5.7%, p < 0.0001) compared to the regular diet group (−2.8 ± 6.2%, *p* = 0.0347; time × group, *p* = 0.0002; Figure 2D). These effects for BMI (*p* = 0.025), body fat percentage (*p* = 0.027), and visceral fat (*p* = 0.028) remained significant after adjusting for baseline differences. The waist-to-hip ratio showed significant reductions in all diet groups (low-fat, −1.3 ± 2.7%; low-carb, −2.0 ± 2.5%; regular diet, −1.1 ± 2.5%; all *p* ≤ 0.0034), without a significant difference over time (time × group, *p* = 0.1022; Figure 2E). Of note, skeletal muscle mass was equally preserved for all diet groups during CR (time × group, *p* = 0.5494; Figure 2C). An analysis based on a more stringent dietary compliance criterion of 80% yielded comparable results for all body composition variables.

### 10-year mortality risk reduction by dietary intervention during CR

**Figure 3:**
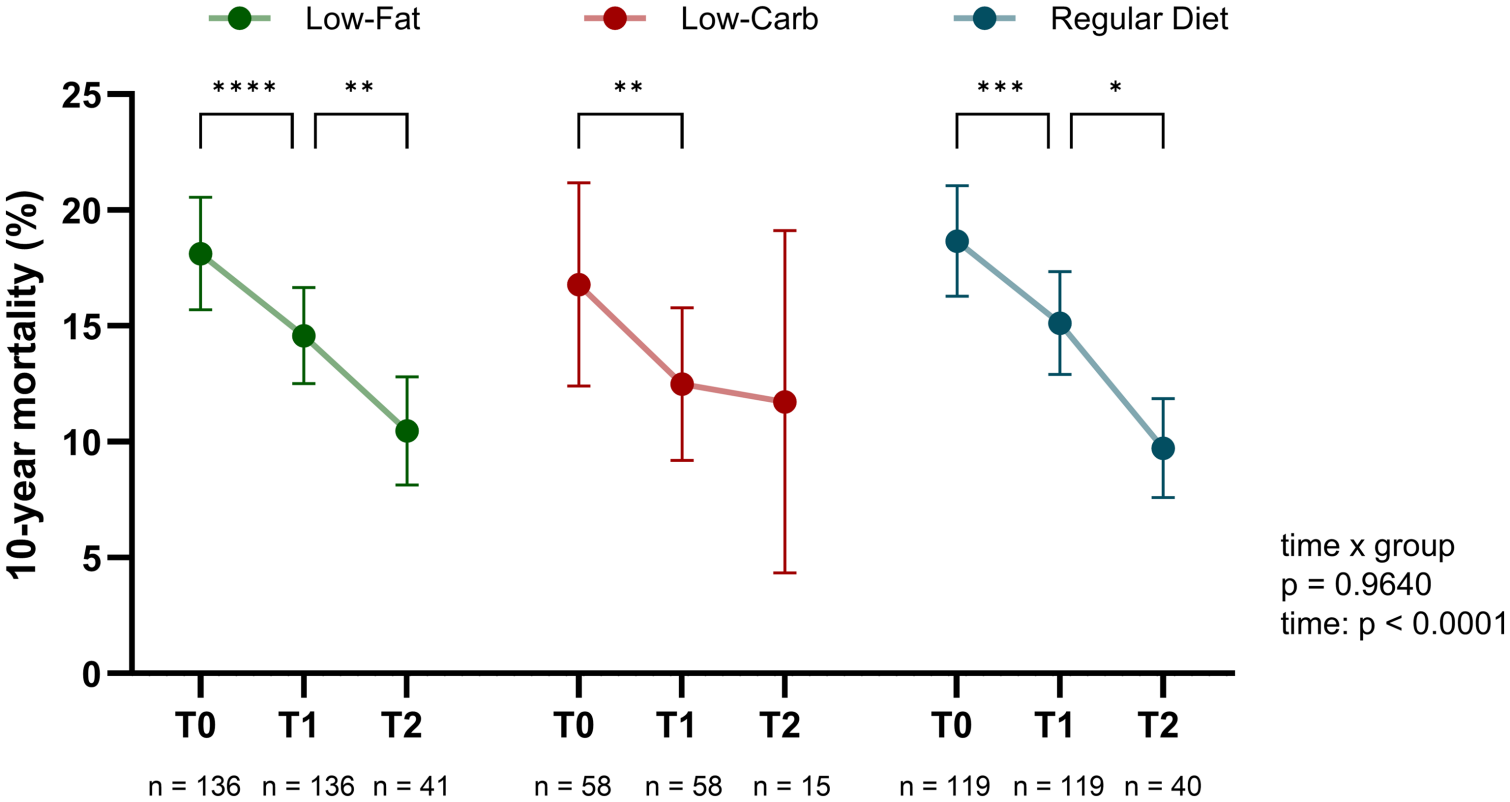
Impact of dietary interventions on cardiovascular risk reduction during and after cardiac rehabilitation (CR). The predicted mortality risk, assessed by the cardiac rehabilitation biomarker score (CRBS), significantly decreased during inpatient CR across all dietary groups. Further reduction was detected in the low-fat and regular diet group during the six-month follow-up, whereas no further reduction was observed in the low-carb group. The predicted 10-year mortality risk (%) is shown at admission (T0), discharge (T1, after 3–4 weeks of inpatient CR), and after 6 months (T2). Data is presented as mean with 95% confidence interval (CI). *p* values indicate between-group comparison over time by mixed-effects model (time × group). ****p < 0.0001, ***p < 0.001, **p < 0.01, *p < 0.05.

At the onset of inpatient CR (T0), the mean 10-year mortality risk indicated by the CRBS score was 18.1% ± 14.4% in the low-fat group, 16.5% ± 16.5% in the low-carb group, and 18.7% ± 13.1% in the regular diet group, without a significant difference between the groups (*p* = 0.694; Figure 3). After 3–4 weeks of CR (T1), a significant reduction in CRBS was observed with a mean overall risk reduction of 3.68 ± 9.58%, which was comparable between dietary groups (low-fat, −3.6 ± 11.1%; low-carb, −4.2 ± 9.4%; regular diet, −3.5 ± 7.6%; all *p* ≤ 0.0030), without a significant difference between the groups (interaction term, time × dietary group, *p* = 0.8651). Of note, a sensitivity analysis using a stricter dietary adherence threshold of ≥ 80% confirmed the findings for CRBS over time between the groups. The Framingham risk score was calculated as a comparative measure of cardiovascular risk and the mean relative risk reduction during inpatient CR was comparable between both scores, with an average overall reduction of ~4.2% in the Framingham score without significant differences between dietary groups (*p* = 0.997).

**Figure 4:**
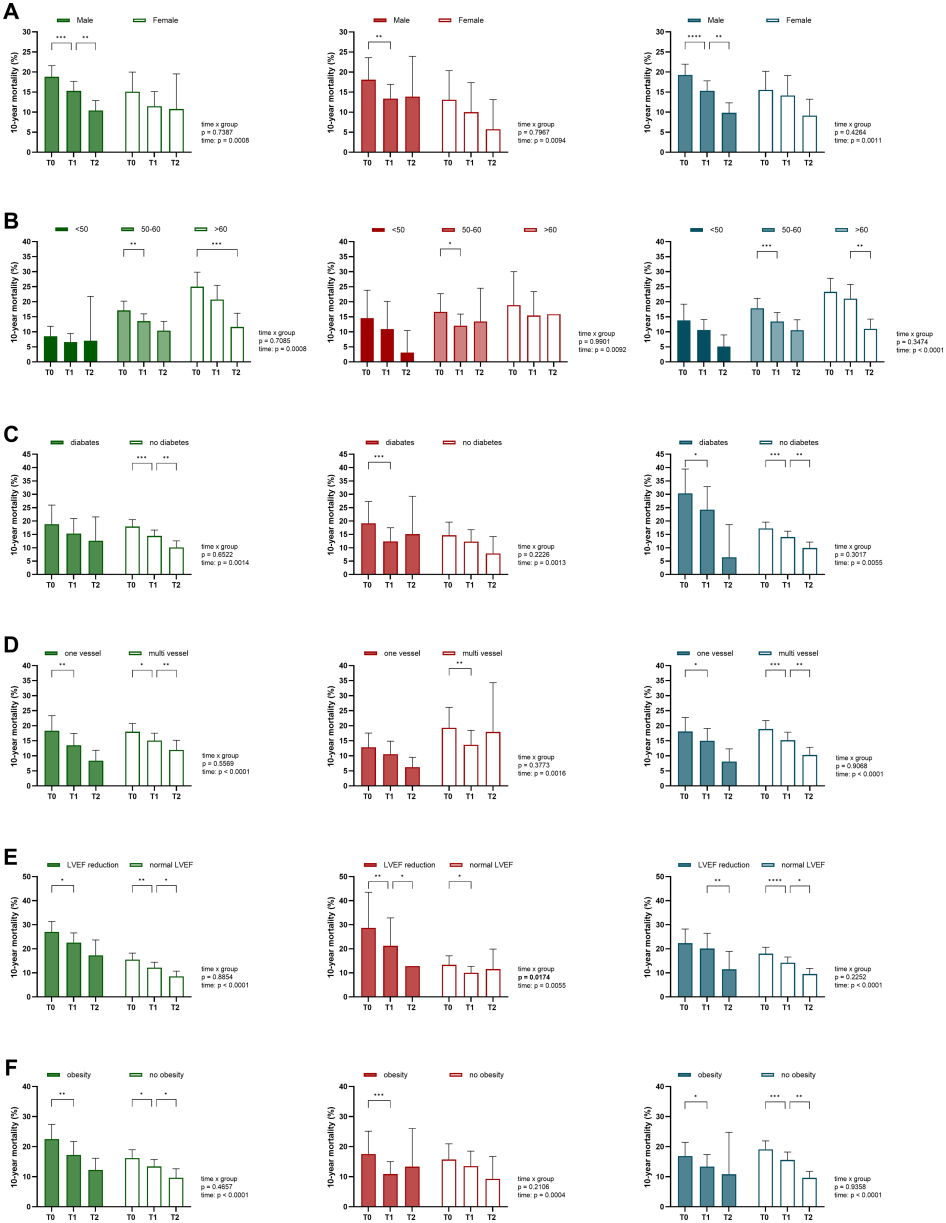
Subgroup-specific risk development across dietary interventions. Subgroup analyses demonstrated that the predicted 10-year mortality risk varied according to patient characteristics across dietary groups (low-fat [green], low-carb [red], and regular diet [blue]). Data is presented for CAD patients grouped by: **(A)** sex, **(B)** age, **(C)** diabetes, **(D)** disease severity, **(E)** LVEF function, and **(F)** obesity. Data is presented as mean with 95% confidence interval (CI). *p* values indicate between-group comparison over time by mixed-effects model (time × group). T0 (admission), T1 (discharge), and T2 (six-month follow-up). ****p < 0.0001, ***p < 0.001, **p < 0.01, *p < 0.05.

To investigate if different effects of the dietary interventions existed depending on age, sex, disease severity and comorbidities including diabetes and obesity, subgroup analyses were performed (Figure 4). In terms of sex, men showed a significant risk reduction across all diets (low-fat, −3.5 ± 12.1%; low-carb, −4.7 ± 10.9%; regular diet, −3.9 ± 7.6%; all *p* ≤ 0.0031), while no significant reduction was observed in women (all *p* ≥ 0.0988; Figure 4A). However, the interaction effects were not significant (interaction term, time × sex, all *p* ≥ 0.1788). In terms of age, patients aged 50–60 years showed a significant reduction across all dietary groups (low-fat, −3.6 ± 12.0%; low-carb, −4.6 ± 10.7%; regular diet, −4.4 ± 6.5%; all *p* ≤ 0.0212), whereas those under 50 and over 60 years did not experience a significant reduction in any group during CR (all *p* ≥ 0.0522; Figure 4B). Diabetic patients exhibited a significant reduction in the low-carb (−6.8 ± 11.6%, *p* = 0.0009) and regular diet group (−6.1 ± 11.5%, *p* = 0.0121), while no significant changes were observed in the low-fat group (−3.5 ± 12.0%, *p* = 0.0995; Figure 4C). However, the risk reduction during CR did not differ significantly between the dietary groups among diabetic patients, even after adjusting for T0 CRBS values (*p* = 0.181). In terms of disease severity, single-vessel disease patients showed a significant reduction in the low-fat group (−4.8 ± 11.4%, *p* = 0.0035) and regular diet group (−3.1 ± 9.4%, *p* = 0.0339), with no significant difference between the dietary interventions (*p* = 0.655; Figure 4D). Multi-vessel disease patients exhibited significant reductions across all diet groups (low-fat, −2.9 ± 10.9%; low-carb, −5.6 ± 10.3%; regular diet, −3.8 ± 6.7%; all *p* ≤ 0.0107). Patients with reduced LVEF during CR (T0 to T1) showed a significant reduction only in the low-fat group (−4.4 ± 9.2%, *p* = 0.0262) and low-carb group (−7.5 ± 11.9%, *p* = 0.006), while the reduction did not differ significantly between the dietary groups (*p* = 0.503; Figure 4E). Regarding obesity, all three dietary groups showed a significant risk reduction in obese patients (low-fat, −5.3 ± 10.3%; low-carb, −6.7 ± 10.9%; regular diet, −3.5 ± 5.9%; all *p* ≤ 0.0452), without a significant difference between dietary interventions (*p* = 0.388; Figure 4F). After adjustment for multiple comparisons using FDR, all subgroup effects that were significant in the unadjusted within-group analysis remained statistically significant. In conclusion, the subgroup analysis did not indicate that one of the three dietary interventions led to a significantly greater risk reduction in a specific patient group.

### Changes in lipids, HbA1c and CRP by dietary intervention during CR

**Figure 5:**
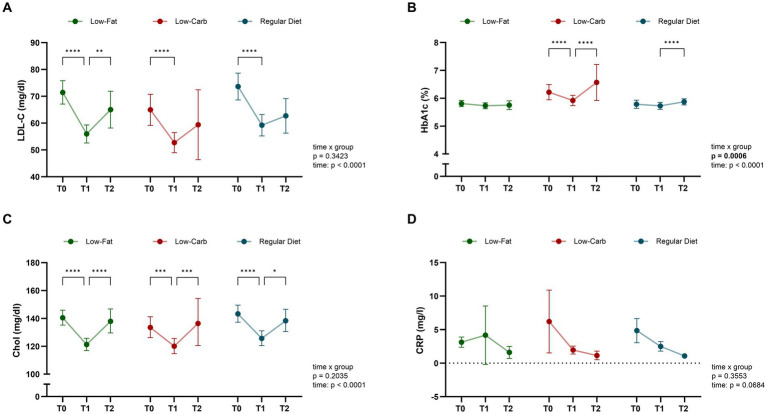
Dynamic of blood biomarkers across dietary interventions. Significant changes in blood lipid profiles and glycemic control were observed for dietary groups (low-fat, low-carb, and regular diet) during inpatient CR. Data is presented for CAD patients grouped by dietary intervention in terms of: **(A)** low-density lipoprotein cholesterol (LDL-C), **(B)** glycated hemoglobin (HbA1c), **(C)** total cholesterol, and **(D)** high-sensitivity C-reactive protein (hsCRP). Data is presented as mean with 95% confidence interval (CI). *p* values indicate within-group comparison over time by mixed-effects model (time × group). T0 (admission), T1 (discharge), and T2 (six-month follow-up). ****p < 0.0001, ***p < 0.001, **p < 0.01, *p < 0.05.

While changes in blood lipid profiles and long-term glycemic control indicated by HbA1c are largely dependent on optimized medical prescription and adherence, dietary interventions may still affect these values to some extent. In this respect, equal improvements in blood lipid profiles were observed from T0 to T1 across all dietary groups (Figure 5). LDL-C levels decreased significantly in all groups during CR (low-fat, −18.8 ± 20.1%; low-carb, −14.3 ± 20.6%; regular diet, −16.7 ± 20.6%; all *p* < 0.0001), with no significant difference over time (time × group, *p* = 0.5510; Figure 5A). Of note, HbA1c levels showed a significant reduction only in the low-carb group (−4.0 ± 6.6%, p < 0.0001) with a significant difference between groups (time × group, *p* = 0.0127), with the low-fat (−1.0 ± 8.4%, *p* = 0.0284) and regular diet group (−0.7 ± 3.8%, *p* = 0.1263) not showing a significant reduction (Figure 5B). However, after adjusting for baseline HbA1c levels, the percentage of diabetic patients, and changes in diabetes medication (both intensification and reduction) using ANCOVA, the difference was no longer statistically significant (*p* = 0.168). Total cholesterol levels also decreased significantly in all diet groups during CR (low-fat, −12.5 ± 14.2%; low-carb, −8.9 ± 14.5%; regular diet, −10.9 ± 15.7%; all *p* < 0.0001), with no significant differences between the dietary groups over time (time × group, *p* = 0.3957; Figure 5C). In terms of inflammation, CRP levels did not show significant changes in any of the diet groups (all *p* ≥ 0.1674; Figure 5D). These results remained consistent even when applying a stricter dietary adherence threshold of 80%.

### Long-term effects on clinical and metabolic parameters by dietary intervention

To investigate the long-term effects of the initial dietary interventions, follow-up visits after 6 months of phase III CR maintenance were performed. Following the initial risk reduction during inpatient CR, overall patients’ risk continued to reduce during phase III CR (T1-T2; Figure 2). This was most prominent for the low-fat (T1, 14.6 ± 12.2%; T2, 10.5 ± 7.4%; *p* = 0.0053) and regular diet (T1, 15.1 ± 12.2%; T2, 9.7 ± 6.7%; *p* = 0.0119) groups, while no significant change was observed in the low-carb group (T1, 12.3 ± 12.3%; T2, 11.7 ± 13.3%; *p* = 0.5044), however, without a significant between-group effect (time × group, *p* = 0.4260). While risk reduction remained stable in most patient subgroups, patients in the low-carb group, particularly men over 50 years of age with diabetes, obesity, and multi-vessel CAD showed a tendency towards increasing risk in the maintenance phase, even though this trend was not significantly different between the dietary groups (all *p* ≥ 0.220; Figure 3). With respect to body composition, BMI, total body fat percentage, visceral fat, and waist-to-hip ratio, a continuous significant decrease was only observed in the low-fat group (all *p* ≤ 0.0457), with no significant difference over time (time × group, all *p* ≥ 0.0842). Of note, while skeletal muscle mass remained stable in the low-fat and regular diet group, skeletal muscle mass increased significantly in the low-carb group (*p* = 0.0348; Figure 2C). In terms of blood parameters, a significant re-increase for total cholesterol levels was observed in all groups (all *p* ≤ 0.0186), while LDL-C increased significantly in the low-fat group (*p* = 0.0015; Figure 5). Of note, HbA1c levels showed a marked increase in the low-carb group and slight increase in the regular diet group (both *p* < 0.0001), which was significant compared to the low-fat group (time × group, *p* = 0.0050).

### Incidence of MACCE in relation to dietary intervention

**Figure 6:**
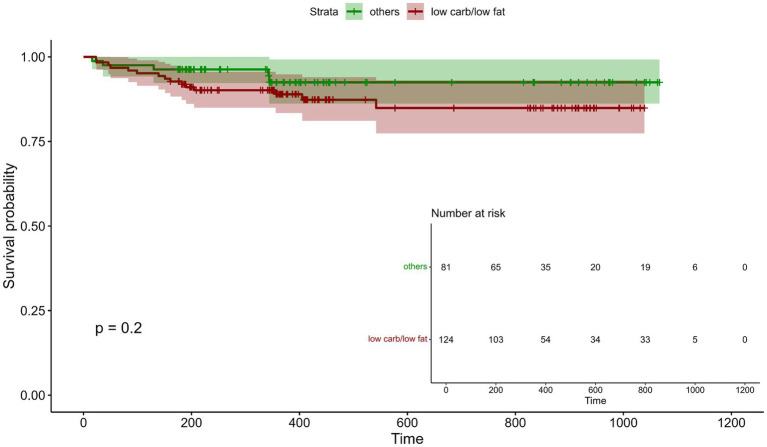
Incidence of major adverse cardiovascular and cerebrovascular events (MACCE) post CR in relation to dietary intervention. Kaplan–Meier curves illustrate the relationship between low-fat/ low-carb diet and regular diet for cardiac rehabilitation biomarker score (CRBS) at the end of CR (*n* = 205) and the occurrence of MACCE during the mean follow-up period of 470 ± 293 days.

To assess whether any of the dietary interventions during CR affected the incidence of MACCE after phase II CR, Kaplan–Meier survival analysis was conducted, comparing patients following a low-carb or low-fat diet with those on a regular diet (Figure 6). Out of 313 patients, follow-up data with a mean time of 470 ± 293 days were available for 205 patients (~ 65%; low-fat, *n* = 87; low-carb, *n* = 37; regular diet, *n* = 81). During the follow-up period, a total of 20 MACCEs were recorded, with 12 events (13.8%) occurring in the low-fat group, 3 (8.1%) in the low-carb group, and 5 (6.2%) in the regular diet group. These included 12 cases of (unplanned) stent placement or in-stent restenosis, 1 CABG, 2 defibrillator implantations, and 3 MIs. Survival analysis did not reveal a significant difference in MACCE incidence between the low-carb/low-fat group and the regular diet group (*p* = 0.2), and these findings were conformed after adjustment for baseline risk using Cox analysis (*p* = 0.538). Visual inspection of Schoenfeld residuals did not indicate violations of the proportional hazards assumption for CRBS (Supplementary Figure 1). A fully adjusted sensitivity model including age, sex, diabetes mellitus, CAD extent, history of MI or bypass surgery, HbA1c, LDL-C, and BMI (at T1) yielded consistent results, with no association between diet group and MACCE incidence (*p* = 0.315). A post-hoc, event-driven power calculation (low-carb/low-fat vs. regular diet) indicated ~80% power for hazard ratios >3.6, indicating that the null finding for MACCE should be interpreted cautiously as smaller but potentially clinically meaningful effects cannot be excluded.

## Discussion

The primary aim of this study was to assess the effects of two dietary strategies—a “low-carb” diet and a “low-fat” diet—on cardiovascular risk reduction in patients with CAD undergoing inpatient CR. Using a biomarker-based risk assessment, we examined both the short-term impact of these interventions during inpatient phase II CR and their long-term effectiveness during the maintenance phase. The key findings of this study are: (1) no difference between patients following a low-carb or low-fat diet in reduction of the 10-year mortality risk was seen during inpatient CR, (2) both dietary interventions resulted in significant reductions in BMI, WHR, body fat and visceral fat, with a tendency of more stable reductions in the low-fat group, (3) diabetic patients in the low-carb group experienced a more pronounced improvement in HbA1c during phase II CR, though this effect was not sustained after discharge, (4) maintenance of low-carb gains post-rehabilitation appeared more challenging, with a tendency towards risk re-elevation, particularly in diabetic and middle-aged patients, and (5) no difference in MACCE for both interventions compared to the regular diet were detected, suggesting that a low-carb and a low-fat diet are safe for CAD patients during and after CR.

Overall, our data suggests that a structured CR program including dietary intervention following evidence-based dietary guidelines results in a significant reduction of the predicted 10-year cardiovascular mortality risk independent of the macronutrient composition. Since neither a low-carb nor a low-fat diet led to a greater additional risk reduction compared to a heart-healthy standard diet, our findings indicate that both dietary approaches were equally effective in risk reduction within the CR setting. While previous studies have demonstrated that both low-carb and low-fat diets can improve metabolic health when applied in a controlled setting (18), others have reported superior long-term effects of low-carb diets, particularly in metabolically healthy individuals, with greater reductions in estimated 10-year cardiovascular risk compared to low-fat diets after 12 months of intervention (mean overall reduction of ~0.6% based on the Framingham score) (12). However, our findings suggest that this advantage does not extend to patients with established CAD undergoing 3 to 4 weeks of inpatient CR. While the controlled intervention phase in our study was shorter, the overall effect of CR including supervised exercise, optimized medical therapy, and comprehensive lifestyle modifications on risk reduction was larger in both applied scores, the CRBS and the Framingham score and may have outweighed any differential impact of dietary macronutrient composition on cardiovascular risk within this patient population. Given the quasi-experimental design with assisted self-selection into dietary arms, potential selection bias needs to be considered when discussing the effects of the different dietary interventions. While a more adverse adiposity and diabetes profile was observed in the low-carb group at baseline, the observed imbalances were mitigated by adjusting the prespecified models for key covariates and, where applicable, the respective baseline value (e.g., HbA1c, lipids, and body composition). Additional checks (including analyses restricted to patients with ≥80% dietary adherence) also yielded results consistent with the primary analyses.

Dietary interventions are a fundamental component of CR, aligning with the ESC guidelines, which advocate for dietary modifications as a cornerstone of secondary prevention (31). While all groups, including the regular diet group, experienced significant cardiovascular risk reduction which underscores the strong impact of CR in general, the low-carb and low-fat intervention demonstrated additional benefits in terms of larger reductions in BMI, body fat, and visceral fat despite comparable muscle mass and likely basal metabolic rate. These findings are in line with previous studies suggesting that both low-carb and low-fat diets can support weight reduction when applied in structured settings (16). A meta-analysis of 59 studies reported modest weight loss benefits for low-carb diets compared to other dietary approaches, particularly over an intervention period of 6 to 12 months, while differences were not statistically significant in shorter or longer-term comparisons. In our study, follow-up data indicated differences in the sustainability of effects in that patients in the low-fat group showed more stable reductions in body composition over time, whereas the low-carb group exhibited a partial reversal of initial benefits. It cannot be excluded that the greater initial weight loss in the low-carb and low-fat group was driven by lower overall caloric intake rather than macronutrient composition, also because caloric intake beyond lunch was not systematically assessed. In addition, as post-discharge adherence was not systematically assessed, between-diet differences should be regarded as hypothesis-generating and interpreted with caution. Nevertheless, this highlights the importance of long-term feasibility and adherence support when evaluating dietary strategies in the context of real-life secondary prevention. In addition, we also observed significant improvements in lipid profiles across all dietary groups during inpatient CR, including reductions in LDL-C and total cholesterol levels. These effects are in line with the expected effects of medication optimization and adherence during CR but were comparable between all dietary groups. During follow-up, a re-increase in lipid levels across all groups and regardless of initial dietary intervention was seen. This rebound effect suggests that lipid lowering therapy during CR maintenance needs close control for all CAD patients, also when considering the lasting weight-lowering effect of dietary interventions, specifically low-fat diets. This phenomenon reflect factors beyond dietary composition, such as adjustments in medication after discharge or declining adherence to dietary recommendations or medication. While low-carb diets have previously been associated with anti-inflammatory effects in some populations (16, 18), particularly through reductions in CRP, we did not observe a significant reduction in CRP levels in any of the dietary groups. The course of CRP remained comparable between the intervention and regular diet group during inpatient CR, suggesting that in the context of established CAD, short-term dietary macronutrient composition may have limited impact on systemic inflammation.

Since safety of specifically low-carb diets has been discussed for CAD patients, we analyzed the occurrence of MACCE following CR with respective dietary intervention over a mean of 460 days. No differences in the incidence of MACCE were observed between the low-carb and low-fat diet groups, and in comparison to the regular diet group during the follow-up period. This finding is most relevant, especially considering ongoing concerns regarding the cardiovascular safety of low-carb diets in high-risk populations. Our results suggest that, when applied in a structured and medically supervised environment, both dietary strategies can be considered safe options for patients with CAD. Regarding patients with a mild to moderate reduction in LVEF, subgroup analyses revealed no significant differences in risk reduction between dietary groups after baseline adjustment. Nevertheless, the numerically larger absolute reduction observed in the low-carb group among patients with reduced LVEF may suggest a potential benefit in this population that warrants further investigation. In interpreting subgroup findings more broadly, it should also be considered that women were underrepresented (~20%) in this trial, which limits generalizability to female patients. Sex-specific differences in cardiovascular risk profiles, adiposity distribution, and differential responses to dietary macronutrient composition as well as to CR have been described (13, 14, 29). Thus, sex-stratified results in this cohort are exploratory and likely underpowered.

Importantly, our findings support the potential of the CRBS as a sensitive tool to track changes in cardiovascular risk during and after rehabilitation, including those related to dietary interventions. Compared to established scores such as the Framingham risk score, the CRBS integrates commonly available biomarkers of cardiac stress, renal function, inflammation, and glycemic control in addition to traditional risk factors. This broader scope allows a more dynamic yet robust assessment of short-term changes during CR, where conventional scores may be less responsive. The CRBS was developed and validated in cohorts of patients with stable CAD (11, 19, 20), supporting its predictive accuracy for long-term outcomes. Nevertheless, its incremental prognostic value in the specific context of CR requires further confirmation in prospective studies. While no significant difference between dietary groups was observed in CRBS during inpatient CR, a group-specific trend emerged during the follow-up period. In particular, only patients in the low-carb group showed no further improvement in CRBS beyond the inpatient setting and values remained essentially unchanged during follow-up. This suggests that the CRBS may be capable of capturing changes in cardiovascular risk associated with adherence to dietary interventions beyond the acute rehabilitation phase. To better understand which components of the CRBS drive these changes, we investigated the development of individual biomarkers over time and found that HbA1c showed a significant short-term improvement in the low-carb group, aligning with previous evidence that carbohydrate restriction can rapidly improve glycemic control in patients with insulin resistance or type 2 diabetes (32, 33). However, after adjusting for baseline HbA1c, diabetes prevalence, and changes in diabetes medication, this effect was not statistically significant, suggesting that the HbA1c reductions were particularly relevant in patients with diabetes. Furthermore, the trend towards increasing HbA1c levels during follow-up in the low-carb group suggests that long-term adherence to carbohydrate restriction may be challenging for many patients. This aligns with prior studies reporting high dropout rates and declining adherence to low-carb diets beyond 6 months, particularly among diabetic patients (33, 34). The difficulty in sustaining a low-carb diet in daily life could be attributed to several factors, including limited availability of suitable meal options, social constraints, and preferences (35). Given the tendency for risk re-elevation in the low-carb group post-rehabilitation, structured follow-up strategies are essential to support adherence. This is particularly relevant for diabetic patients, who may benefit from a more flexible dietary approach that balances carbohydrate moderation with long-term feasibility. To this end, one potential strategy is the integration of personalized nutritional counseling into CR maintenance programs. Regular follow-up appointments, smartphone-based dietary tracking and goal achievement combined with tailored patient education could enhance adherence and reinforce dietary modifications over time. Additionally, motivational approaches such as shared decision-making and goal setting may help improve patient engagement and long-term dietary adherence (9, 36–38). Future research should explore the effectiveness of these interventions in maintaining dietary benefits beyond the acute rehabilitation phase.

### Limitations

First, the quasi-experimental, self-selected assignment to diet groups entails potential selection bias and although prespecified covariate adjustment and sensitivity checks were performed, residual confounding cannot be excluded. Propensity score methods were not performed. Furthermore, while the study included a relatively large sample of CAD patients, the proportion of female participants was low (~20%), which may limit the generalizability of findings to women. In addition, follow-up data was available for only about two-thirds of the cohort. While comparative analyses did not reveal systematic differences in baseline characteristics or medication use between participants with and without follow-up, which reduces the likelihood of attrition bias, the possibility of residual bias from unmeasured factors cannot be excluded. Objective post-discharge dietary adherence was not assessed, and total daily energy intake in the post-rehabilitation phase was not quantified. Accordingly, long-term conclusions should be considered as hypothesis-generating. The analysis of MACCE is limited by the low number of events, resulting in limited statistical power and an increased risk of type II error, so moderate between-diet differences cannot be excluded.

## Conclusion

This study provides valuable insights into the role of dietary interventions in CR. While both low-carb and low-fat diets were associated with a significant reduction in cardiovascular risk during inpatient rehabilitation, differences in their sustainability after discharge became apparent through follow-up risk trajectories. The low-carb diet was associated with significant short-term improvements in glycemic control, particularly in diabetic patients, but adherence challenges limited its sustainability post-rehabilitation. In contrast, the low-fat diet demonstrated greater long-term stability in body weight and lipid regulation. Since no difference in MACCE was detected, the dietary interventions may be considered equally safe for CAD patients.

## Data Availability

The raw data supporting the conclusions of this article will be made available by the authors, without undue reservation.
