# Supplementary material for: Biomarker-based risk assessment of dietary intervention in patients with coronary artery disease during cardiac rehabilitation—a quasi-experimental study
Source: Front Nutr. 2025 Oct 23;12:1669931. doi: 10.3389/fnut.2025.1669931 (PMC12590557; doi:10.3389/fnut.2025.1669931)
Supplement: Supplementary file 1 [file Data_Sheet_1.pdf]

Biomarker-based risk assessment of dietary intervention in patients with coronary artery disease during cardiac rehabilitation – a quasi-experimental study

Kotewitsch et al.

Table of Contents

1. Supplementary Table 1: Therapeutic characteristics.
2. Supplementary Figure 1: Schoenfeld residuals for CRBS at T1 in the Cox proportional hazards model.

Supplementary Table 1: Therapeutic characteristics.

	Overall (n = 282)	Low-Carb (n = 53)	Low-Fat (n = 115)	Regular diet (n = 114)	P-value
Completed therapy units (n)	49.5 ± 12.7	51.0 ± 13.0	50.2 ± 13.1	48.0 ± 12.1	0.267
Metabolic equivalents (METs)	109 ± 38.5	105 ± 38.9	112 ± 39.6	109 ± 37.3	0.601
Cardiorespiratory METs	95.7 ± 34.7	90.9 ± 35.3	96.9 ± 35.8	96.7 ± 33.3	0.545
Strength training METs	13.7 ± 11.5	14.2 ± 10.5	14.7 ± 11.6	12.4 ± 11.7	0.317
Total energy expenditure (kcal)	10430 ± 4198	10801 ± 4291	10874 ± 4483	9808 ± 3797	0.113

METs were calculated according to the compendium of physical activities by Ainswoth et al. Data were available for 90% of participants. Data is presented as mean values per stay ± SD. Kcal, kilocalorie. P-values indicate between-group comparison by ANOVA.

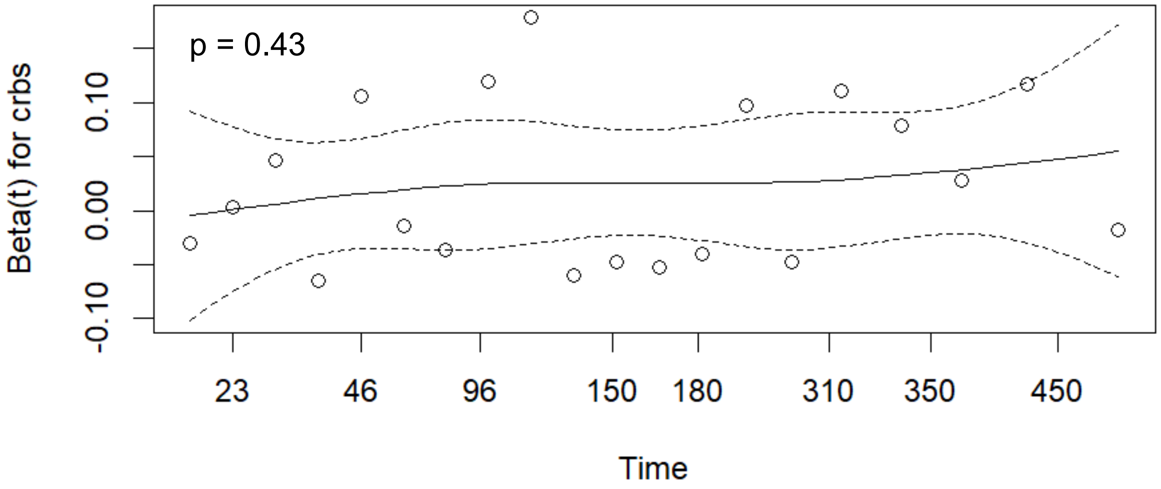

Supplementary Figure 1: Schoenfeld residuals for CRBS at T1 in the Cox proportional hazards model. Circles represent scaled Schoenfeld residuals at each event time, the continuous line depicts a smoothed estimate of the time-dependent coefficient, and dashed lines represent 95% confidence bands. The absence of systematic deviation from zero indicates that the proportional hazards assumption was not violated. The x-axis indicates time in days during follow-up observation.
